# Deterministic Quantum Dense Coding Based on Non-Maximal Entangled Channel

**DOI:** 10.3390/e27020104

**Published:** 2025-01-22

**Authors:** Xuanxuan Xin, Zhixing Li, Zhen Wang

**Affiliations:** 1 College of Arts and Science, Qingdao Binhai University, Qingdao 266555, China; 2 Dalian University of Technology and Belarusian State University Joint Institute, Dalian University of Technology, Dalian 116024, China; 3 School of Physics and Electronic Technology, Liaoning Normal University, Dalian 116029, China

**Keywords:** quantum communication, quantum dense coding, non-maximally entangled channel

## Abstract

In quantum communication, the concept of dense coding traditionally relies on maximally entangled states as quantum channels. Recent advancements have expanded this framework to include non-maximally entangled states within the probabilistic dense coding paradigm. However, such schemes introduce a significant limitation: the receiver cannot always retrieve the complete dense coding information sent by the sender. Consequently, the receiver must inform the sender of the amount of information successfully received. Based on this feedback, the sender determines whether retransmission is necessary, leading to inefficient use of the quantum channel and reduced communication efficiency. To address these shortcomings, we propose an alternative deterministic quantum dense coding scheme that utilizes non-maximally entangled states as the quantum channel. This deterministic approach eliminates the need for retransmissions and significantly enhances communication efficiency while maintaining compatibility with non-maximally entangled states. Our scheme represents a substantial improvement over existing probabilistic methods and paves the way for more efficient quantum communication protocols.

## 1. Introduction

With the rapid advancement of quantum information science [1], the field of quantum communication has experienced significant progress, underpinned by both theoretical breakthroughs and experimental implementations [2,3,4,5,6,7,8]. Central to these advancements is the phenomenon of quantum entanglement [9,10,11,12,13], a uniquely quantum resource that plays a foundational role in enabling various quantum communication protocols. Entanglement serves as the critical enabler for transformative technologies such as quantum dense coding [3,14,15,16,17], which enhances communication efficiency by encoding classical information into quantum states. Additionally, it facilitates quantum teleportation [18,19,20,21,22], a protocol that transfers quantum states across spatially separated systems, and quantum secure direct communication (QSDC) [23,24,25,26], which ensures the direct and secure transfer of information without requiring a shared secret key. Together, these protocols exemplify the transformative potential of entanglement-based quantum communication, highlighting its critical role in shaping the future of secure and efficient information exchange. In the conventional quantum dense coding scheme [3], it is imperative for the two communicating parties to establish a maximally entangled channel, i.e., a shared maximally entangled state, to ensure deterministic transmission of information. However, the preparation of maximally entangled states in practice remains a formidable challenge [27,28,29]. While it is possible to establish a general entanglement channel between the two communicating parties using current experimental techniques, the unavoidable environmental noise and system decoherence make it difficult to establish and store a maximally entangled channel under harsh conditions [30,31,32,33,34]. Since non-maximally entangled states are easier to prepare and store relative to ideal maximally entangled states, research on quantum communication has expanded from maximally entangled channels to non-maximally entangled channels [35,36,37,38,39,40,41]. In traditional quantum dense coding schemes based on non-maximal entanglement channels [38,42,43,44,45,46], due to the fact that the channel between Sender Alice and Receiver Bob is not built by the maximally entangled state (Bell state), Bob cannot directly measure the quantum state of the qubit reserved by Bob and qubit sent by Alice. He needs to perform unitary operations to transform the quantum state of these two qubits with the help of auxiliary qubits. To let Alice make sure that he has lost information and that she needs to send the lost information again, Bob must report the measurement result of the auxiliary qubit.

To address the issue of probabilistic dense coding, we have provided here an alternative deterministic quantum dense coding scheme based on a non-maximally entangled channel. In this deterministic communication scheme, we design a set of unitary operations encoded with 00,01,10, and 11. Then, using the auxiliary qubit and specific unitary operations performed by Alice, Bob can directly obtain two bits of information encoded in quantum states. Regardless of whether the quantum channel used is an ideal maximally entangled state or a more easily prepared and stored non-maximally entangled state, there is no theoretical probability of failure for dense coding. Our approach contributes to making quantum communication more efficient and easier to use in practical quantum communication scenarios by increasing the utilisation of quantum entanglement and reducing the associated overhead. In addition, this scheme offers the potential for wider applications in quantum communication networks, where maximizing entanglement utilization and minimizing quantum resource cost are essential for scalable and reliable information transmission.

## 2. Deterministic Quantum Dense Coding on Non-Maximum Entangled State

This section presents a detailed account of our quantum dense coding scheme. Let us consider the scenario in which Alice and Bob share a non-maximally entangled pure state. The qubit designated as A is in the possession of Alice, while the qubit designated as B is in the possession of Bob. The quantum state of the entanglement is given by

(1) $${|\psi_{1}\rangle }_{AB}=\alpha {|00\rangle }_{AB}+\beta {|11\rangle }_{AB}$$

where the coefficients α and β are both complex, with the modulus of α less than that of β, i.e., 0<|α|<|β|. Additionally, the two coefficients satisfy the following normalization condition: ${\left|\alpha \right|}^{2}+{\left|\beta \right|}^{2}=1$. It is assumed here that the communicating parties have verified the legitimacy of the received information through the quantum identity authentication process [47,48,49,50,51].

### 2.1. Unitary Operations

In this section, we introduce the unitary operations and orthogonal basis employed in this deterministic communication scheme. It is similar to the initial quantum dense coding scheme and probabilistic dense coding scheme in that Alice will perform four different unitary operations on her qubit, but the difference is that Alice needs to add an auxiliary qubit *e* with the initial quantum state ${|0\rangle }_{e}$. The joint quantum state employed in this scheme becomes

(2) $${|\psi_{0}\rangle }_{ABe}={|\psi_{1}\rangle }_{AB}{|0\rangle }_{e}=\alpha {|00\rangle }_{AB}{|0\rangle }_{e}+\beta {|11\rangle }_{AB}{|0\rangle }_{e}.$$

It can be demonstrated that the joint bases of the quantum state of qubit *A*, *B*, and *e* are the bases ${|000\rangle }_{eAB}$, ${|101\rangle }_{eAB}$, ${|010\rangle }_{eAB}$, ${|111\rangle }_{eAB}$, ${|100\rangle }_{eAB}$, ${|001\rangle }_{eAB}$, ${|110\rangle }_{eAB}$, and ${|011\rangle }_{eAB}$. Alice is capable of executing four operations on the qubit *A* and the auxiliary qubit *e* she adds. These four unitary operations, as outlined in this scheme, are comprised of three unitary matrices as follows:

(3) $$U=\left(\begin{array}{cccc} \frac{x}{\alpha }\sin\theta & \frac{y}{\alpha }\cos\theta & 0 & 0\\ \frac{y}{\alpha }\cos\theta & -\frac{x}{\alpha }\sin\theta & 0 & 0\\ 0 & 0 & \frac{x}{\beta }\cos\theta & \frac{y}{\beta }\sin\theta \\ 0 & 0 & -\frac{y}{\beta }\sin\theta & \frac{x}{\beta }\cos\theta \end{array}\right),$$

(4) $$U_{x,e}=I_{A}⊗\sigma_{x}\left(e\right)⊗I_{B},$$

(5) $$U_{x,A}=\sigma_{x}\left(A\right)⊗I_{\alpha }⊗I_{B},$$

where

(6) $$\left\{\begin{array}{c} x=\sqrt{\frac{\beta^{2}-\sin^{2}\theta }{1-2\sin^{2}\theta }}\\ y=\sqrt{\frac{\alpha^{2}-\sin^{2}\theta }{1-2\sin^{2}\theta }} \end{array}.\right.$$

α and β are the coefficients associated with the entanglement channel, and θ is the coefficient associated with the measured basis vectors. These three coefficients need to be disclosed, as with conventional quantum-dense coding schemes. To ensure that the values of *x* and *y* are reasonable, a necessary condition is that the absolute value of sinα must be less than the lesser of the modes of α and β. In addition, the matrix *U* is unitary, i.e., $UU^{†}=1$. The four unitary operations, designated as *U*, $UU_{x,e}$, $U_{x,A}U$, and $U_{x,A}UU_{x,e}$, are applied in sequence to the quantum state $|\psi_{0}\rangle_{ABe}$, and the resulting four states are

(7) $$\begin{array}{cc} U{|\psi_{0}\rangle }_{ABe} & =x{\left(\sin\theta |00\rangle +\cos\theta |11\rangle \right)}_{AB}{|0\rangle }_{e}\\ \; & +y{\left(\cos\theta |00\rangle -\sin\theta |11\rangle \right)}_{AB}{|1\rangle }_{e}, \end{array}$$

(8) $$\begin{array}{cc} UU_{x,e}{|\psi_{0}\rangle }_{ABe} & =y{\left(\cos\theta |00\rangle -\sin\theta |11\rangle \right)}_{AB}{|0\rangle }_{e}\\ \; & +x{\left(\sin\theta |00\rangle +\cos\theta |11\rangle \right)}_{AB}{|1\rangle }_{e}, \end{array}$$

(9) $$\begin{array}{cc} U_{x,A}U{|\psi_{0}\rangle }_{ABe} & =x{\left(\sin\theta |10\rangle +\cos\theta |01\rangle \right)}_{AB}{|0\rangle }_{e}\\ \; & +y{\left(\cos\theta |10\rangle -\sin\theta |01\rangle \right)}_{AB}{|1\rangle }_{e}, \end{array}$$

(10) $$\begin{array}{cc} U_{x,A}UU_{x,e}{|\psi_{0}\rangle }_{ABe} & =y{\left(\cos\theta |10\rangle -\sin\theta |01\rangle \right)}_{AB}{|0\rangle }_{e}\\ \; & +x{\left(\sin\theta |10\rangle +\cos\theta |01\rangle \right)}_{AB}{|1\rangle }_{e}. \end{array}$$

### 2.2. Orthogonal Complete Basis

In order to distinguish the aforementioned quantum states and realize deterministic dense coding, we have designed the following orthogonal basis for orthogonal measurement:

(11) $$\left\{\begin{array}{c} {|\psi_{1}\rangle }_{AB}=\sin\theta {|00\rangle }_{AB}+\cos\theta {|11\rangle }_{AB}\\ {|\psi_{2}\rangle }_{AB}=\cos\theta {|00\rangle }_{AB}-\sin\theta {|11\rangle }_{AB}\\ {|\psi_{3}\rangle }_{AB}=\sin\theta {|10\rangle }_{AB}+\cos\theta {|01\rangle }_{AB}\\ {|\psi_{4}\rangle }_{AB}=\cos\theta {|10\rangle }_{AB}-\sin\theta {|11\rangle }_{AB} \end{array}\right.$$

Obviously, the four quantum states displayed in Equation (11) are mutually orthogonal and collectively form the basis for a four-dimensional Hilbert space. We can define an operator $T_{ij}$ as follows:

(12) $$\begin{array}{cc} T_{ij}= & -2{|\psi_{1}\rangle }_{ijij}\langle \psi_{1}|-{|\psi_{2}\rangle }_{ijij}\langle \psi_{2}|\\ \; & +{|\psi_{3}\rangle }_{ijij}\langle \psi_{3}|+2{|\psi_{4}\rangle }_{ijij}\langle \psi_{4}|. \end{array}$$

By performing the operator on the quantum state of qubit *A* and qubit *B*, Bob can deduce the quantum state from the Hamilton value obtained. When the Hamilton values are −2, −1, 1, and 2, the quantum states are ${|\psi_{1}\rangle }_{AB}$, ${|\psi_{2}\rangle }_{AB}$, ${|\psi_{3}\rangle }_{AB}$, and ${|\psi_{4}\rangle }_{AB}$, respectively.

### 2.3. Process of Dense Coding

Alice introduces an auxiliary qubit *e*, designated as $|\psi_{0}\rangle_{e}$, into the entangled state, represented as $|\psi_{1}\rangle_{AB}$, which is shared between herself and Bob. The entangled state can be demonstrated as follows: ${|\psi_{1}\rangle }_{AB}{|0\rangle }_{e}$. Subsequently, Alice selects one of the unitary operations from the set *U*, $UU_{x,e}$, $U_{x,A}U$, or $U_{x,A}UU_{x,e}$, and applies it to qubit *A* and qubit *e*. Consequently, the quantum state is transformed as follows:

(13) $$\left\{\begin{array}{c} U{|\psi_{1}\rangle }_{AB}∼x{|\psi_{1}\rangle }_{AB}{|0\rangle }_{e}+y{|\psi \rangle }_{AB}{|1\rangle }_{e}\\ UU_{x,e}{|\psi_{1}\rangle }_{AB}∼x{|\psi_{1}\rangle }_{AB}{|1\rangle }_{e}+y{|\psi_{2}\rangle }_{AB}{|0\rangle }_{e}\\ U_{x,A}U{|\psi_{1}\rangle }_{AB}∼x{|\psi_{3}\rangle }_{AB}{|0\rangle }_{e}+y{|\psi_{4}\rangle }_{AB}{|1\rangle }_{e}\\ U_{x,A}UU_{x,e}{|\psi_{1}\rangle }_{AB}∼x{|\psi_{3}\rangle }_{AB}{|1\rangle }_{e}+y{|\psi_{4}\rangle }_{AB}{|0\rangle }_{e}, \end{array}\right.$$

Next, Alice performs an orthogonal measurement on qubit *e*. If the measurement is ${|0\rangle }_{e}$, the state will collapse into

(14) $$\begin{array}{cc} U{|\psi_{1}\rangle }_{AB} & ∼x{|\psi_{1}\rangle }_{AB}{|0\rangle }_{e}+y{|\psi_{2}\rangle }_{AB}{|1\rangle }_{e}\\ & ∼{|\psi_{1}\rangle }_{AB}{|0\rangle }_{e}, \end{array}$$

(15) $$\begin{array}{cc} UU_{x,e}{|\psi_{2}\rangle }_{AB} & ∼x{|\psi_{1}\rangle }_{AB}{|1\rangle }_{e}+y{|\psi_{2}\rangle }_{AB}{|0\rangle }_{e}\\ & ∼{|\psi_{2}\rangle }_{AB}{|0\rangle }_{e}, \end{array}$$

(16) $$\begin{array}{cc} U_{x,A}U{|\psi_{3}\rangle }_{AB} & ∼x{|\psi_{3}\rangle }_{AB}{|0\rangle }_{e}+y{|\psi_{4}\rangle }_{AB}{|1\rangle }_{e}\\ & ∼{|\psi_{3}\rangle }_{AB}{|0\rangle }_{e}, \end{array}$$

(17) $$\begin{array}{cc} U_{x,A}UU_{x,e}{|\psi_{4}\rangle }_{AB} & ∼x{|\psi_{3}\rangle }_{AB}{|1\rangle }_{e}+y{|\psi_{4}\rangle }_{AB}{|0\rangle }_{e}\\ \; & ∼{|\psi_{4}\rangle }_{AB}{|0\rangle }_{e}. \end{array}$$

If the measurement is ${|1\rangle }_{e}$, the state will collapse into

(18) $$\begin{array}{cc} U{|\psi_{1}\rangle }_{AB} & ∼x{|\psi_{1}\rangle }_{AB}{|0\rangle }_{e}+y{|\psi_{2}\rangle }_{AB}{|1\rangle }_{e}\\ & ∼{|\psi_{2}\rangle }_{AB}{|1\rangle }_{e}, \end{array}$$

(19) $$\begin{array}{cc} UU_{x,e}{|\psi_{2}\rangle }_{AB} & ∼x{|\psi_{1}\rangle }_{AB}{|1\rangle }_{e}+y{|\psi_{2}\rangle }_{AB}{|0\rangle }_{e}\\ & ∼{|\psi_{1}\rangle }_{AB}{|1\rangle }_{e}, \end{array}$$

(20) $$\begin{array}{cc} U_{x,A}U{|\psi_{3}\rangle }_{AB} & ∼x{|\psi_{3}\rangle }_{AB}{|0\rangle }_{e}+y{|\psi_{4}\rangle }_{AB}{|1\rangle }_{e}\\ & ∼{|\psi_{4}\rangle }_{AB}{|1\rangle }_{e}, \end{array}$$

(21) $$\begin{array}{cc} U_{x,A}UU_{x,e}{|\psi_{4}\rangle }_{AB} & ∼x{|\psi_{3}\rangle }_{AB}{|1\rangle }_{e}+y{|\psi_{4}\rangle }_{AB}{|0\rangle }_{e}\\ & ∼{|\psi_{3}\rangle }_{AB}{|1\rangle }_{e}. \end{array}$$

Alice then sends qubit A to Bob, along with the measurement result of qubit e via a classical channel. In order to perform an orthogonal measurement on the quantum state of qubit A and qubit B, Bob utilizes the basis defined in Equation (11), which comprises the four quantum states: $\left\{{|\psi_{1}\rangle }_{AB},{|\psi_{2}\rangle }_{AB},{|\psi_{3}\rangle }_{AB},{|\psi_{4}\rangle }_{AB}\right\}$. The measurement result allows Bob to ascertain precisely which operation Alice has performed. In this process, Bob can obtain two bits of information. To illustrate, if the measurement outcome for qubit A and qubit B is found to be the state vector of the form $|\psi_{1}\rangle_{AB}$ and the measurement outcome for qubit e is found to be the state vector of the form ${|0\rangle }_{e}$, then it can be concluded that the operation performed by Alice is the unitary operator *U*. In the event that the measurement result of qubit A and qubit B is represented by the state vector $|\psi_{4}\rangle_{AB}$ and the measurement result of qubit *e* is represented by the state vector ${|1\rangle }_{e}$, it follows that Bob will conclude that the operation performed by Alice is the composite operation $U_{x,A}U$. As can be seen from Table 1, the information that Bob can obtain in different situations is dependent on the encoded value, which is represented by the digits 00, 01, 10, and 11. The values represented by these digits are *U*, $UU_{x,e}$, $U_{x,A}U$, and $U_{x,A}UU_{x,e}$.

### 2.4. Discussion

Prior to the formal dense encoding, a quantum identity authentication process allows for defending against man-in-the-middle (MITM) attacks as well as for verifying the legitimacy of the receiver. During the dense coding process, even if an eavesdropper has access to the measurements of the auxiliary particles, they will not be able to access the information to be delivered. This is because the eavesdropper is not aware of the operations performed by the sender in relation to the auxiliary particles. From the process outlined above, it is evident that in deterministic dense coding, if we take ${|\psi_{1}\rangle }_{AB}=\sin\theta {|00\rangle }_{AB}+\cos\theta {|11\rangle }_{AB}$ as the quantum channel, dense coding is performed optimally when the value of the angle parameter, represented by the variable θ, is within the range of 0 to α. The specific value of α can be determined as needed. In a classical communication of this process, only the measurement results of the auxiliary qubit are transferred. It is therefore necessary to ensure that third parties cannot directly obtain the encoded information by listening to the measurement results of the auxiliary qubit. We now turn to consider the optimal range of the variable θ from the perspective of information security. In this case, regardless of the value of the non-maximal state of α that constitutes the quantum channel, and irrespective of the degree of entanglement of the entangled states, two bits of information can be transferred with each communication, provided that the appropriate unitary operation is selected. It is required that the value of θ in the unitary operation Equation (5) and basis Equation (11) satisfies the condition of 0<θ<arcsinα. As the value of θ decreases, the value of $x^{2}$, where $x^{2}=\frac{\cos^{2}\theta -\alpha^{2}}{1-2\sin^{2}\theta }$, increases. In the event that the value of $x^{2}$ is excessive and the third eavesdropper, Crosen, is in receipt of the measurement result pertaining to the auxiliary qubit between Alice and Bob, Crosen is able to infer the following from Equation (10): In the event that the measurement result of the auxiliary qubit is 0, it will correspond with high probability to the unitary operation *U* and $U_{x,A}U$. Similarly, when the measurement result of the auxiliary qubit is 1, it will correspond with high probability to the unitary operations $UU_{x,e}$ and $U_{x,A}UU_{x,e}$. It is evident that information disclosure has occurred in these instances. Therefore, in order to guarantee information security, it is imperative that the value of θ is not excessively diminished. Furthermore, the entangled state value of α in the quantum channel should not be too small either, given that θ is less than arcsinα.

## 3. Conclusions

In conclusion, this work extends deterministic quantum dense coding to non-maximally entangled channels, marking a significant advancement over traditional probabilistic dense coding protocols using non-maximally entangled states. The proposed scheme ensures the deterministic transmission of two bits of classical information per communication, effectively addressing the limitations of probabilistic approaches. A key advantage of this method is that it eliminates the need for the receiver to report the measurement results of the auxiliary qubit back to the transmitter, thereby reducing the communication overhead. Additionally, the scheme requires only a single unitary operation by the sender during the entire communication process, in contrast to probabilistic schemes that demand separate operations by both the sender and receiver for each communication round. These improvements streamline the communication process, reduce resource consumption, and enhance the overall efficiency of quantum information transfer. This deterministic approach not only optimizes the use of quantum channels but also lays a foundation for more practical and scalable implementations of quantum dense coding in future quantum communication networks.

## Figures and Tables

**Table 1:** Different measurements correspond to different coded information.

Orthogonal Measurement	Measurement of Auxiliary Qubit *e*	Unitary Matrix	Coded Message
${|\psi_{1}\rangle }_{AB}$	${|0\rangle }_{e}$	*U*	00
${|\psi_{1}\rangle }_{AB}$	${|1\rangle }_{e}$	$UU_{x,e}$	01
${|\psi_{2}\rangle }_{AB}$	${|0\rangle }_{e}$	$UU_{x,e}$	01
${|\psi_{2}\rangle }_{AB}$	${|1\rangle }_{e}$	*U*	00
${|\psi_{3}\rangle }_{AB}$	${|0\rangle }_{e}$	$U_{x,A}U$	10
${|\psi_{3}\rangle }_{AB}$	${|1\rangle }_{e}$	$U_{x,A}UU_{x,e}$	11
${|\psi_{4}\rangle }_{AB}$	${|0\rangle }_{e}$	$U_{x,A}UU_{x,e}$	11
${|\psi_{4}\rangle }_{AB}$	${|1\rangle }_{e}$	$U_{x,A}U$	10

## Data Availability

The data that support the findings of this study are available from the corresponding author(s) upon reasonable request.
